# Intravenous tranexamic acid vs. sublingual misoprostol in high-risk women for postpartum haemorrhage following cesarean delivery; a randomised clinical trial

**DOI:** 10.1186/s12884-023-05935-5

**Published:** 2023-08-25

**Authors:** Mariam Dawoud, Maha Al-Husseiny, Omneya Helal, Moutaz Elsherbini, Mazen Abdel-Rasheed, Mona Sediek

**Affiliations:** 1 Obstetrics and Gynaecology Department, Faculty of Medicine, Cairo University, Cairo, Egypt; 2 Reproductive Health Research Department, National Research Centre, 33 El-Buhouth St, Dokki, 12622 Cairo, Egypt

**Keywords:** Misoprostol, Oxytocin, Postpartum haemorrhage, Tranexamic acid

## Abstract

**Objective:**

This study compares the effectiveness of administering sublingual misoprostol combined with oxytocin to that of IV tranexamic acid combined with oxytocin to reduce intra and post-operative blood loss in high-risk women for postpartum haemorrhage (PPH) following cesarean section (CS).

**Methods:**

About 315 high-risk pregnant women undergoing CS participated in this trial. They were randomly assigned into three groups; tranexamic group, misoprostol group, and control group, according to the medication given in the operative theatre. All patients received oxytocin intraoperatively. They were assessed regarding intraoperative blood loss, the incidence of PPH, and the reduction in haemoglobin and hematocrit values.

**Results:**

Both tranexamic and misoprostol groups had similar results in reducing intra and post-operative blood loss. However, the reduction in haemoglobin and hematocrit were significantly lower in tranexamic and misoprostol groups compared to the control group (-0.78 ± 0.57 vs. -0.83 ± 0.52 vs. -1.32 ± 0.57 gm/dl, P < 0.001 and − 3.05 ± 1.28 vs. -3.06 ± 1.13 vs. -4.94 ± 1.82%, P < 0.001 respectively). In addition, the estimated blood loss was significantly lower in the tranexamic and misoprostol groups compared to the control group (641.6 ± 271.9 vs. 617.9 ± 207.4 vs. 1002.4 ± 340.7 ml, P < 0.001).

**Conclusion:**

Both tranexamic acid and misoprostol are equally capable of reducing blood loss, but the results were significantly better compared to using oxytocin alone in high-risk patients.

**Clinical Trial Registration:**

Registered at www.clinicaltrials.govon07/10/2019 with registration number NCT04117243.

## Introduction

The cesarean section (CS) rate is still sharply growing, as CS is the commonest major obstetric procedure performed worldwide [1]. Despite the advances in the medical field, obstetric haemorrhage remains a well-recognised complication of childbirth in both developed and developing countries [2, 3]. Obstetric haemorrhage is identified as the second leading cause of maternal mortality in developed countries while considered the primary cause of maternal mortality in developing countries [4, 5].

Postpartum haemorrhage (PPH), either primary or secondary, is considered one of the commonest types of obstetric haemorrhage. In 2017, the American College of Obstetrics and Gynecology updated the definition of primary PPH to be a cumulative blood loss higher than 1000 mL with clinical features of hypovolemia within 24 h of birth, regardless of the delivery route [6]. Uterine atony, lacerations, retained tissues or blood clots and coagulation factor deficiencies are the most common causes of PPH. The management strategies include uterine massage, oxytocin, methylergometrine, and circulatory support with or without blood transfusion. It has been estimated that about 5% of cesarean delivery may experience PPH [7, 8].

Since prevention of PPH is the cornerstone of management, the National Collaborating Centre for Women’s and Children’s Health has recommended the administration of intravenous 5 IU of oxytocin routinely following the cesarean delivery as a prophylactic measure against PPH [9].

Several studies have assessed the use of other agents in addition to oxytocin for the prophylaxis against PPH following CS. Misoprostol, a prostaglandin E1 analogue, has been introduced as a uterotonic agent to prevent PPH after CS. A Cochrane review has concluded that the combination of misoprostol and oxytocin was one of the most effective combinations in reducing blood loss compared to oxytocin alone [10]. In addition, WHO has issued a statement recommending the distribution of misoprostol among pregnant women in low-source countries to be used after delivery to reduce blood loss [11].

Tranexamic acid is an antifibrinolytic medication that acts by blocking lysine binding sites on plasminogen molecules. Several studies have addressed its use in preventing PPH following CS and showed the effectiveness of tranexamic acid when added to oxytocin in preventing blood loss [12, 13]. A Cochrane review has also shown its effectiveness when used alone in a dose of 0.5-1 gm intravenously in low-risk women for PPH. However, it was concluded that further studies were required to assess its safety profile and its use in high-risk women [14].

Our study aimed to reach the most effective protocol in reducing intra and post-operative blood loss in high-risk women for PPH following CS. Therefore, we compared the effectiveness of the combined use of sublingual misoprostol and IV oxytocin with that of the combined use of IV tranexamic acid and oxytocin. Also, we compared them with the effectiveness of oxytocin when given alone.

## Methods

A randomised clinical trial was carried out, following the CONSORT guidelines, in Kasr Al-Ainy Hospital (Obstetrics and Gynaecology Department, Faculty of Medicine, Cairo University) from January 2020 to December 2020 after approval of the Medical Ethical Committee. Informed consent was obtained from all participants after explaining the nature of the study, expected value, outcome, and possible adverse effects. This clinical trial was registered at www.clinicaltrials.govon07/10/2019 with registration number NCT04117243.

The study included 345 pregnant women who were candidates for lower segment cesarean section (LSCS) under spinal anaesthesia. Inclusion criteria were maternal age 20–40 years, term pregnancy (≥ 37 weeks), with one or more of the high risk for PPH criteria [15]. These criteria included: (1) maternal anaemia (haemoglobin < 9.9 g%), (2) chronic maternal medical disorders (e.g., cardiac, renal, DM), (3) preeclampsia or gestational hypertension, (4) macrosomia, (5) high-risk cases for obstetric haemorrhage (e.g., peripartum haemorrhage, accidental haemorrhage, placenta previa, previous history of uterine atony or PPH).

On the other hand, exclusion criteria were (1) intrauterine fetal death (IUFD), (2) fetal anomalies or growth retardation (FGR), (3) emergency CS, (4) more than two previous CS procedures, (5) prolonged procedure (more than two hours from skin incision to skin closure), (6) abnormally invasive placenta, (7) known or history of thromboembolic events, (8) history of prostaglandin or Tranexamic acid allergy.

All participants underwent the following steps to confirm their eligibility for this study: (1) full medical and obstetric history, (2) general and obstetric examination, (3) obstetric ultrasound, and (4) pre-operative laboratory tests: including complete blood count (CBC), coagulation profile, and liver and kidney function tests.

On the day of the scheduled surgery, the participants were randomly assigned into three groups; Tranexamic Group, Misoprostol Group, and Oxytocin-only Group (as a control group). Randomisation was performed using computer-generated random numbers.

In the tranexamic group, 1 gm (10 ml) of tranexamic acid (Kapron, Amoun, Egypt) was diluted in 20 ml of Glucose 5%, then given to the patients as an intravenous infusion over 5 min, at least 15 min before skin incision. In the misoprostol group, 400 micrograms of misoprostol (2 tablets - Cytotec, Pfizer, G.D. Searle LLC) were administered sublingually by the patients immediately before starting the skin incision.

Following the baby’s delivery, all patients in the three groups received an intravenous bolus of 5 IU oxytocin (Syntocinon, Novartis, Basel, Switzerland) and 20 IU oxytocin in 500 mL lactated Ringer’s solution (infused at a rate of 125 mL/h). The operative time was recorded, the blood volume in the suction unit was observed, and the number of operative towels was counted.

All patients were observed for primary PPH for the first 24 h. They were also followed regarding the occurrence of misoprostol-related side effects (shivering, pyrexia > 38 C, headache, nausea, and vomiting in the first 6 h) and the occurrence of tranexamic acid-related side effects (thromboembolic events within one week of delivery).

CBC was repeated 12 h after delivery, and the estimated blood loss (EBL) after CS was calculated by this formula:

$$EBL\, = \,EBV \times \frac{{{\rm{Pre}} - {\rm{operative}}\,{\rm{hematocrit}} - {\rm{Postoperative}}\,{\rm{hematocrit}}}}{{{\rm{Pre}} - {\rm{operative}}\,{\rm{hematocrit}}}},$$

where EBV is the estimated blood volume of the patient in mL = weight in kg × 85 [16].

The primary outcome was to compare the estimated blood loss (EBL) during and after cesarean delivery among the three groups, while the secondary outcomes were to evaluate the incidence of PPH and the possible side effects.

### Sample size calculation

The sample size was calculated with PASS 11 software (NCSS, LLC. Kaysville, Utah, USA). The sample size of 95 for each group achieves 90% power to detect a difference of 100.8 between the null hypothesis and the alternative hypothesis that their means are 499.9 and 600.7 with estimated group standard deviations of 206.4 and 215.7 and with a significance level (alpha) of 0.05 using a two-sided two-sample t-test [17]. The sample size was increased by 20% to be 114 for each group to allow for dropouts.

### Statistical methods

Recorded data were analysed using the statistical package for the Social Sciences (SPSS) version 25. Quantitative variables were summarised in the form of mean and standard deviation, while categorical variables were summarised in the form of numbers and percentages. The numerical data were compared with a one-way analysis of variance (ANOVA) when comparing between means and with the Kruskall-Wallis test if the data were non-parametric. For comparing the categorical data, a Chi-square (x^2^) test was performed. P values less than 0.05 were considered statistically significant.

## Results

**Fig. 1:**
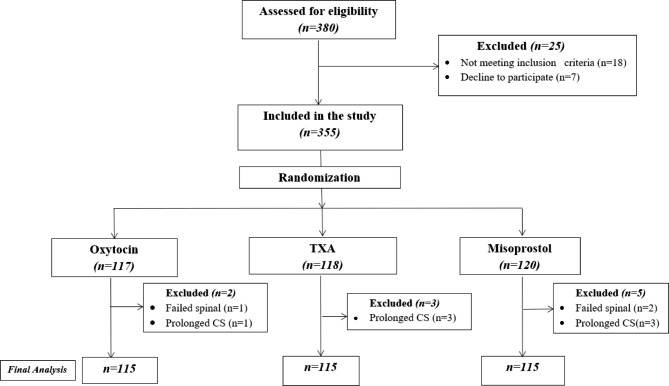
Flow diagram of patients in the study

In this clinical trial, 345 pregnant women met the inclusion criteria and assigned to three groups, as shown in the flowchart of patients in Fig. 1. The demographic and clinical characteristics of the participants are demonstrated in Table 1. All groups had no significant difference regarding maternal age, BMI, parity, indication for CS, gestational age at delivery, pre-operative Hb and HCT, and operative time.

The maternal outcomes are shown in Tables 2 and 3. Both tranexamic and misoprostol groups had similar results regarding the post-operative Hb and HCT, the reduction in Hb and HCT values, the blood loss in the suction apparatus and the EBL. There were no significant differences between both groups.

**Table 1:** Basic demographic and clinical characteristics of the participants

	Tranexamic Group (n = 115)	Misoprostol Group (n = 115)	Control Group (n = 115)	P-value
**Maternal age (years)**	29.59 ± 4.15	28.70 ± 4.51	29.90 ± 5.15	0.125
**BMI (kg/m2)**	30.56 ± 3.60	30.54 ± 4.30	30.19 ± 3.44	0.903
**Parity** - **Primigravida** - **Para 1** - **Para 2 or more**	7 (6.09%) 13 (11.30%) 95 (82.61%)	8 (6.96%) 9 (7.83%) 98 (85.22%)	7 (6.09%) 18 (15.65%) 90 (78.26%)	0.480
**GA at delivery**	38.46 ± 0.97	38.50 ± 0.96	38.38 ± 0.95	0.622
**CS Indication** - **previous CS** - **CPD** - **Abnormal presentation** - **Placenta Previa** - **ICSI**	86 (74.8%) 3 (2.6%) 11 (9.6%) 12 (10.4%) 3 (2.6%)	86 (74.8%) 5 (4.3%) 9 (7.8%) 11 (9.6%) 4 (3.5%)	76 (66.1%) 8 (7.0%) 15 (10.4%) 14 (12.2%) 2 (1.7%)	0.667
**Pre-operative Hb (gm/dl)**	11.17 ± 0.89	11.42 ± 1.05	11.21 ± 1.11	0.146
**Pre-operative HCT (%)**	34.20 ± 2.64	34.94 ± 3.42	34.70 ± 3.24	0.188
**Estimated blood volume (ml)**	7169 ± 546	7121 ± 719	7108 ± 556	0.726
**CS Duration (minutes)**	73.88 ± 14.95	77.19 ± 11.12	74.24 ± 15.26	0.142
**Interval from skin incision to complete fetal and placental extraction (minutes)**	15.15 ± 1.14	15.10 ± 0.89	14.95 ± 1.38	0.385

**Table 2:** Maternal outcomes in Caesarean section

	Tranexamic Group (n = 115)	Misoprostol Group (n = 115)	Control Group (n = 115)	P-value
**Number of soaked towels**	5 (2–10)	4 (2–9)	6 (2–10)	< 0.001*
**Blood loss in suction apparatus (ml)**	247.4 ± 115.6	248.7 ± 93.5	395.2 ± 142.2	< 0.001*
**Post-operative Hb (gm/dl)**	10.39 ± 0.87	10.58 ± 1.03	9.89 ± 1.07	< 0.001*
**Hb difference (gm/dl)**	-0.78 ± 0.57	-0.83 ± 0.52	-1.32 ± 0.57	< 0.001*
**Post-operative HCT (%)**	31.15 ± 2.62	31.88 ± 3.38	29.76 ± 3.07	< 0.001*
**HCT difference (%)**	-3.05 ± 1.28	-3.06 ± 1.13	-4.94 ± 1.82	< 0.001*
**Estimated blood loss (ml)**	641.6 ± 271.9	617.9 ± 207.4	1002.4 ± 340.7	< 0.001*
**Incidence of postpartum haemorrhage in 1st 24 h**	2 (1.74%)	1 (0.87%)	3 (2.61%)	0.601
**Side effects**	1 (0.9%)	0 (0.0%)	0 (0.0%)	0.367

**Table 3:** Comparison between the three groups regarding

	Groups		Mean Difference (X-Y)	P-value	95% Confidence Interval	
					Lower	Upper
**Post-operative Hb (gm/dl)**	Control (X)	Tranexamic (Y)	-0.50	< 0.001*	-0.808	-0.192
		Misoprostol (Y)	-0.70	< 0.001*	-1.004	-0.388
	Tranexamic (X)	Control (Y)	0.50	< 0.001*	0.192	0.808
		Misoprostol (Y)	-0.20	0.294	-0.504	0.112
	Misoprostol (X)	Control (Y)	0.70	< 0.001*	0.388	1.004
		Tranexamic (Y)	0.20	0.294	-0.112	0.504
**Hb difference (gm/dl)**	Control (X)	Tranexamic (Y)	-0.54	< 0.001*	-0.708	-0.363
		Misoprostol (Y)	-0.49	< 0.001*	-0.659	-0.314
	Tranexamic (X)	Control (Y)	0.54	< 0.001*	0.363	0.708
		Misoprostol (Y)	0.05	0.779	-0.123	0.222
	Misoprostol (X)	Control (Y)	0.49	< 0.001*	0.314	0.659
		Tranexamic (Y)	-0.05	0.779	-0.222	0.123
**Post-operative HCT (%)**	Control (X)	Tranexamic (Y)	-1.39	**0.002**	-2.333	-0.446
		Misoprostol (Y)	-2.12	< 0.001*	-3.063	-1.175
	Tranexamic (X)	Control (Y)	1.39	**0.002**	0.446	2.333
		Misoprostol (Y)	-0.73	0.165	-1.673	0.214
	Misoprostol (X)	Control (Y)	2.12	< 0.001*	1.175	3.063
		Tranexamic (Y)	0.73	0.165	-0.214	1.673
**HCT difference (%)**	Control (X)	Tranexamic (Y)	-1.89	< 0.001*	-2.342	-1.446
		Misoprostol (Y)	-1.89	< 0.001*	-2.334	-1.438
	Tranexamic (X)	Control (Y)	1.89	< 0.001*	1.446	2.342
		Misoprostol (Y)	0.01	0.999	-0.440	0.456
	Misoprostol (X)	Control (Y)	1.89	< 0.001*	1.438	2.334
		Tranexamic (Y)	-0.01	0.999	-0.456	0.440
**Estimated blood loss (ml)**	Control (X)	Tranexamic (Y)	360.74	< 0.001*	274.219	447.260
		Misoprostol (Y)	384.43	< 0.001*	297.914	470.955
	Tranexamic (X)	Control (Y)	-360.74	< 0.001*	-447.260	-274.219
		Misoprostol (Y)	23.70	0.795	-62.825	110.216
	Misoprostol (X)	Control (Y)	-384.43	< 0.001*	-470.955	-297.914
		Tranexamic (Y)	-23.70	0.795	-110.216	62.825
**Blood loss in suction apparatus (ml)**	Control (X)	Tranexamic (Y)	147.83	< 0.001*	110.948	184.704
		Misoprostol (Y)	146.52	< 0.001*	109.644	183.399
	Tranexamic (X)	Control (Y)	-147.83	< 0.001*	-184.704	-110.948
		Misoprostol (Y)	-1.30	0.996	-38.182	35.573
	Misoprostol (X)	Control (Y)	-146.52	< 0.001*	-183.399	-109.644
		Tranexamic (Y)	1.30	0.996	-35.573	38.182

^a^ P-value is significant (ANOVA test with Tukey Post Hoc test)

Unlike that, the post-operative Hb and HCT values were significantly higher in the tranexamic and misoprostol groups compared to the control group (P < 0.001). Subsequently, the reduction in Hb and HCT values was significantly lower in tranexamic and misoprostol groups compared to the control (P < 0.001). In addition, blood loss in the suction apparatus and EBL were significantly lower in the tranexamic and misoprostol groups than in the control group (P < 0.001). However, there was no significant difference between all groups regarding the incidence of PPH in the first 24 h and the side effects.

## Discussion

In this study, the combined use of sublingual misoprostol and IV oxytocin was equally effective as the combined use of IV tranexamic acid and oxytocin in decreasing blood loss in high-risk women undergoing CS. Meanwhile, compared to using oxytocin alone, both protocols were superior in reducing the amount of blood loss.

Hemapirya L et al. (2020) reached similar results, although they included 200 low-risk women candidates for LSCS, who were randomised equally randomised into two groups; the study group in which tranexamic acid was given before skin incision at a dose of 10 ml/kg in 100 ml saline, and a control group which was given the standard 10 IU oxytocin intravenously following the delivery of the baby. The study group had less blood loss and higher post-operative haemoglobin when compared to the control group [18].

In a meta-analysis by Simonazzi et al. (2016) that included 2365 women from nine trials, the pre-operative use of tranexamic acid was associated with lower blood loss, less haemoglobin drop and lower incidence of PPH; when compared to the control who had oxytocin alone [19]. Another systematic review came with the same results regarding the effect of tranexamic acid on decreasing peripartum blood loss. However, only minor side effects were reported following its use, such as shivering and nausea, with no increased risk of thromboembolism. Yet, the authors had safety concerns over the use of tranexamic acid. They explained that the trial was of moderate quality [20].

Regarding the role of adding sublingual misoprostol to oxytocin in preventing PPH, previous studies revealed similar results to our findings [21, 22]. Chaudhuri and Majumdar (2015) studied the effect of sublingual misoprostol in a dose of 400 mcg versus placebo in 198 women undergoing emergency CS and at high risk for blood loss. In their study, misoprostol was given following delivery of the baby, unlike in our study, in which misoprostol was given before skin incision. They also used 20 U of oxytocin IV following delivery of the baby in both groups, whereas we used 10 U of oxytocin. The misoprostol group showed a significantly lower mean intraoperative blood loss compared to the placebo group; however, the post-operative blood loss was slightly lower in the misoprostol group. Side effects such as shivering and pyrexia were reported more in the misoprostol group [21].

In a former study, Fekih et al. (2009) compared the role of sublingual misoprostol administration (in a dose of 200 mcg) at cord clamping together with oxytocin at a dose of 20 U (10 U bolus dose and 10 U infusion in 500 ml lactated Ringer), with that of giving oxytocin alone at the same dose in 250 low-risk women undergoing elective CS. The combined misoprostol and oxytocin group showed less blood loss and less haemoglobin drop than the oxytocin-only group. Again, the combined misoprostol and oxytocin group showed more adverse effects, such as shivering and pyrexia [22].

Although we found that pre-operative use of sublingual misoprostol was equally effective as that of intravenous tranexamic acid to prevent PPH in high-risk women undergoing CS, a previous study by Tabatabaie et al. (2021) revealed that misoprostol is more effective than tranexamic acid in reducing the blood loss intra- and post-operatively [23]. The possible explanation for misoprostol superiority is that they enrolled their study on a non-risk population. On the contrary, Bose and Beegum (2017) found that tranexamic acid is superior to misoprostol in reducing blood loss in non-risk women. However, they found tranexamic acid and misoprostol equally effective in reducing blood loss in high-risk women, which agrees with our results.

The strength of our study is comparing the effectiveness and safety of sublingual misoprostol to that of IV tranexamic acid, as well as to that of oxytocin alone in preventing PPH IN high-risk pregnant women undergoing CS. Our randomised study had a large sample size, and we used different methods to evaluate the effectiveness of each management protocol. However, the main limitation is that our study was open-label, and our population had various risk factors. Also, we did not study the effect of different doses of misoprostol.

## Conclusion

In clinical practice, both IV tranexamic acid and sublingual misoprostol, when used along with oxytocin, are equally capable of reducing blood loss. However, the results were significantly better than using oxytocin alone in high-risk patients. Further studies in the future are needed, especially in low-risk patients, due to the discrepancy in the results of the previous studies.

## Data Availability

The data that support the findings of this study are available from Kasr El-Ainy Hospital, but restrictions apply to the availability of these data, which were used under license for the current study, and so are not publicly available. Data are, however, available from the authors upon reasonable request and with permission of Kasr El-Ainy Hospital.

## Declarations

### Clinical Trial Registration

The clinical trial was registered at www.clinicaltrials.govon07/10/2019 with registration number NCT04117243.
